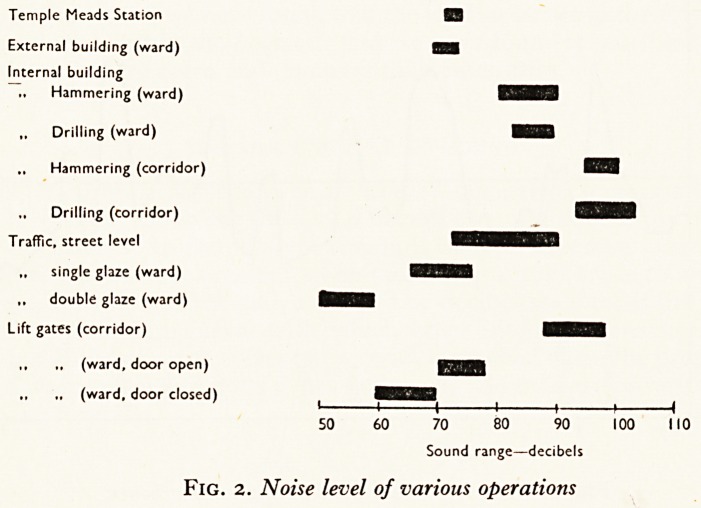# Direct Measurement of Noise Levels in a Large Hospital

**Published:** 1963-01

**Authors:** A. P. Cole, P. H. Dimambro


### DIRECT MEASUREMENT OF NOISE LEVELS IN A LARGE HOSPITAL

BY

A. P. COLE

*Sixth year Medical Student*

AND

P. H. DIMAMBRO, B.SC., GRAD. I.E.E.

Previous studies on the question of noise in hospitals have been based on the replies of patients to questionnaires. This paper records what is to our knowledge the first attempt to determine sound levels in a hospital by direct measurement; the work was carried out in the Bristol Royal Infirmary.

Some justification is needed for the use of objective sound measurement. Admittedly the intensity of a noise is not always directly related to its annoyance value, nevertheless its measurement has great practical value, especially for purposes of comparison. The direct measurement of low-intensity sound helps little—for example even a small sound can be very disturbing at dead of night—but direct measurement of high-intensity sound is vital for a full assessment of the problem. The questionnaires already undertaken fail to indicate how high many of the hospital noise levels are. Contrary to the idea expressed by some that direct measurement would only reveal unavoidable traffic noises, we have found that the loudest noises in the Royal Infirmary are in fact produced within the hospital; and we have found it possible to isolate the various noises and to measure and compare their intensities.

### THE MEASUREMENT OF SOUND

The human ear does not respond in a "linear" manner to increases in the sound energy that it receives. Doubling the sound energy does not double the impression of loudness to the listener. In fact, the ear responds more or less on a logarithmic scale. For this reason it is most convenient when measuring sound levels to work with the decibel system, for this utilizes such a scale. The decibel scale takes the threshold of hearing as the fundamental level with which other intensities are compared. This threshold, for a sound of 1,000 cycles per second, is reached when the pressure exerted by the sound waves reaches 0·0002 dynes per sq.cm. Sounds are compared according to the following formula:

$$\frac{L_A}{L_B} = 20 \log_{10} \frac{P_A}{P_B}$$

where $L$ is the subjective loudness and $P$ is the physical pressure of sounds $A$ and $B$. The comparison is such that when sound levels are high, a great increase in the physical energy of the sound is registered as a rise of relatively few decibels, and *vice versa* when the original sound is low.

The ear also has a variable sensitivity to the intensity of a sound which depends on the sound's pitch. Since the microphone and measuring equipment that we used had no dependence on pitch, electronic circuits were included which compensated for this variation, and the measuring circuit had an overall response very much like that of the human ear. The equipment being transistorized was very light and portable. It used a crystal microphone and recorded decibels directly. It was calibrated by comparison with a standard instrument. Curves called "weighing curves" have been worked out which correct for pitch variations, and we used those of the National Physical Laboratory (Hughes, 1961). Special circuits called "correcting circuits" were included in the instrument which made the necessary corrections for 60, 80 and 100 decibels.

Various attempts have been made to correlate a sound's intensity with its nuisance value, but none of these systems is so established as to warrant its use in this investigation. We had to be satisfied with measuring the background noise level, the variations imposed on this, and the sudden peaks that might be so great that we believe they must indeed have nuisance value.

### RESULTS

### *Environmental noise in the wards*

Figure 1 shows the general sound level in a ward, and some of the peaks mentioned above. The graph does not in fact refer to any one particular ward, but gives a composite picture of the peak and background noises actually measured in six different wards facing the main roads. The values are therefore averages, though in fact all the wards were very much alike.

FIG. 1. *Noise levels in a ward during waking hours.*

Certain well-marked peaks show up which correspond to different events in the routine of the ward. The most prominent correspond to meal times, when the clatter of cutlery and serving out were measured, and when above all the noise of washing up in the all-metal basins occurred. The slamming of the sluices also shows up clearly.

All of this takes place against a continuous background of noise due to traffic, most noticeable in the early morning, during rest periods, and in the evening when the ward itself is quiet, although it is always present. The line across the graph labelled "station" represents the background noise level that we measured in the subway of Temple Meads, Bristol's main station, during a rush hour, and it is included for the purpose of comparison.

### INDIVIDUAL NOISES

1. *Trolleys.* After we had examined 50 per cent of the trolleys in the hospital, it was evident that there had been a decided improvement in their design. There were basically three types: wooden trolleys with large wheels, which were quiet, glass-topped trolleys which were by themselves quiet when properly maintained, and the older metal-topped trolleys which could be extremely noisy. For the purpose of comparison we held a parade of trolleys (bearing sample loads) over the two basic types of hospital floor surface, hard sheen and linoleum. The conclusion was that careful laying out of instruments and unhurried use of the trolley were both necessary to keep the sound at a reasonable level. The trolleys were always noisy when crossing the ridge created by a change of floor surface at a doorway, because everything bounced.

2. *Staff.* We enjoyed the fullest cooperation in our investigation from the nursing staff, and we are happy to say that the noise the staff made was very little, in fact less than that produced by the patients. A houseman's pair of shoes was the only noticeably noisy item contributed by the medical staff. The domestics, however, accounted for a large part of the noise produced by members of the hospital staff. Working as they often do outside the wards, it is doubtful whether they appreciate how much of the noise they make is audible inside.

3. *Building noises.* (Fig. 2.) These were easily the worst of all the noises measured. The loudest drilling and hammering produced readings of 43 to 55 decibels (equivalent to the noise of a pneumatic drill), and these were measured inside the door of a ward on a corridor on which the work was going on. With the doors wide open the noise

FIG. 2. *Noise level of various operations*

within was the same as that without, and with the doors closed it fell to a level of 38 decibels (equivalent to a noisy bus starting up). This noise level was extremely irritating, and both patients and staff were noticeably disturbed. Nor were these intensities momentary; they lasted several hours.

External building noises were at times 26 decibels when the windows were closed. They were produced by operations associated with the installation of a lift-shaft.

4. *The lift.* Next to internal construction, the greatest single source of noise in the hospital was a lift with expanding metal doors. We recorded the noise produced by 50 consecutive gate movements, and many times obtained readings that momentarily reached 50 decibels. Within a neighbouring ward, with closed doors, the loudest lift sound was found to be 24 decibels.

5. *Traffic.* The B.R.I. is sited on either side of a busy main road and the traffic was a persistent cause of a general noise level in wards facing the road. Of 50 individual sounds measured at street level, the loudest recorded 43 decibels. The greatest noises were made by the screeching of car brakes and by buses starting up from either side of a pedestrian crossing, and by motorcycles. Measurements were then taken in a first floor ward beneath an ordinary window (closed) and showed a maximum of 22 decibels, whereas those taken under a closed double-glazed window showed a maximum of 12 decibels.

Nearly all progressive offices on main roads have introduced double glazing on their exterior windows so that the staff can work in peace. The effect, partially demonstrated in this investigation, is dramatic.

### DISCUSSION

Our measurements have revealed that the sounds produced within and around a hospital are indeed so loud that they are bound to distress patients by virtue of their intensity. We discovered, for example, that a noise equivalent to that of a pneumatic drill is heard *inside* the hospital during building operations. This might be unavoidable and only very temporary, but there were comparable permanent sounds. The old-fashioned lift-gates, when slammed hard, produced sounds nearly as great, and these gates have been fixtures for years. We have also shown that at several times during the day the noise level in the wards exceeds that in the subway of Temple Meads Station during a rush hour.

Normally sound intensity diminishes as the square of the distance from the source. But this does not apply in narrow enclosed spaces since the sound is reflected from walls if they are surfaced by non-absorbent plaster. At certain frequencies the reverse effect is very pronounced and booming occurs. The easiest way to overcome this difficulty is to divide up the space by partitions whose surfaces do not reflect sound easily. We soon found that the corridors were much noisier than the wards. When a ward's door is open, the noise level in the ward rises to that in the corridor. Since this happens frequently throughout the day, it is clear that a lower noise level in the corridors would be advantageous. This can be achieved by the use of acoustic tiles, and the isolation of the noisiest parts of the corridors by swinging doors.

The problem of traffic noise remains a perennial one, but measurements show that by far the greatest sounds are actually produced within the hospital, and these could be reduced considerably. Well fitting automatically closing doors, well designed and adequately maintained trolleys, and the use of double glazing have already produced great improvement. But they have to be universally applied to be fully effective.

There can be no doubt that patients are frequently subjected to an incredible amount of noise. Whilst their comfort in every other way is being attended to, they occupy wards that may be noisier than railway stations, with drilling and hammering nearby, and with a background of heavy traffic and noisy hospital equipment. These problems are by no means confined to hospitals, and suitable sound-proofing devices are well known in civil engineering, and could be applied as successfully to hospitals as to other buildings. The writers feel that both in hospital reconstruction and in new building a scientific consideration of this problem is necessary.

### REFERENCES

"Noise Control in Hospital". King Edward VII Hospital Fund. 1958, 1960.

"Electronic Engineers' Reference Book." Hughes, 1961.

## Figures and Tables

**Fig. 1. f1:**
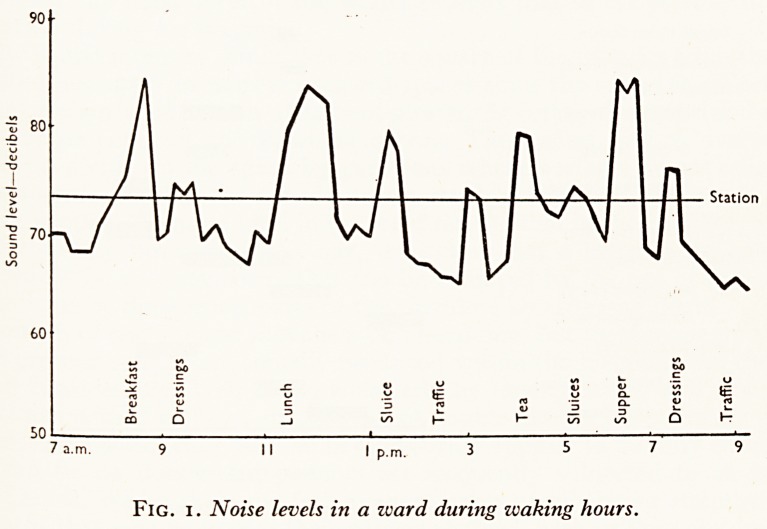


**Fig. 2. f2:**